# Trehalose Protects Maize Plants from Salt Stress and Phosphorus Deficiency

**DOI:** 10.3390/plants8120568

**Published:** 2019-12-04

**Authors:** Md. Motiar Rohman, Md. Robyul Islam, Mahmuda Binte Monsur, Mohammad Amiruzzaman, Masayuki Fujita, Mirza Hasanuzzaman

**Affiliations:** 1 Molecular Breeding Lab, Plant Breeding Division, Bangladesh Agricultural Research Institute, Gazipur 1701, Bangladesh; irobyul@gmail.com (M.R.I.); mahmudamumu0@gmail.com (M.B.M.); amiruzzaman95@yahoo.com (M.A.); 2 Laboratory of Plant Stress responses, Faculty of Agriculture, Kagawa University, Kagawa 7610795, Japan; 3 Department of Agronomy, Faculty of Agriculture, Sher-e-Bangla Agricultural University, Sher-e-Bangla Nagar, Dhaka 1207, Bangladesh

**Keywords:** abiotic stress, oxidative stress, salinity, nutrient deficiency, osmolytes, methylglyoxal

## Abstract

This study is undertaken to elucidate the role of trehalose (Tre) in mitigating oxidative stress under salinity and low P in maize. Eight-day-old maize seedlings of two maize varieties, BARI Hybrid Maize-7 and BARI Hybrid Maize-9, were subjected to salinity (150 mM NaCl), low P (5 µM KH_2_PO_4_) and their combined stress with or without 10 mM Tre for 15 d. Salinity and combined stress significantly inhibited the shoot length, root length, and root volume, whereas low P increased the root length and volume in both genotypes. Exogenous Tre in the stress treatments increased all of the growth parameters as well as decreased the salinity, low P, and combined stress-mediated Na^+^/K^+^, reactive oxygen species (ROS), malondialdehyde (MDA), lipoxygenase (LOX) activity, and methylglyoxal (MG) in both genotypes. Individually, salinity and low P increased superoxide dismutase (SOD) activity in both genotypes, but combined stress decreased the activity. Peroxidase (POD) activity increased in all stress treatments. Interestingly, Tre application enhanced the SOD activity in all the stress treatments but inhibited the POD activity. Both catalase (CAT) and glutathione peroxidase (GPX) activity were increased by saline and low P stress while the activities inhibited in combined stress. Similar results were found for ascorbate peroxidase (APX), glutathione peroxidase (GR), and dehydroascorbate reductase (DHAR) activities in both genotypes. However, monodehydroascorbate reductase (MDHAR) activity was inhibited in all the stresses. Interestingly, Tre enhanced CAT, APX, GPX, GR, MDHAR, and DHAR activities suggesting the amelioration of ROS scavenging in maize under all the stresses. Conversely, increased glyoxalase activities in saline and low P stress in BHM-9 suggested better MG detoxification system because of the down-regulation of glyoxalase-I (Gly-I) activity in BHM-7 in those stresses. Tre also increased the glyoxalase activities in both genotypes under all the stresses. Tre improved the growth in maize seedlings by decreasing Na^+^/K^+^, ROS, MDA, and MG through regulating antioxidant and glyoxalase systems.

## 1. Introduction

Salinity is considered the most destructive abiotic stress that limits crop growth and yield. Under saline stress, plant accumulates salt to a toxic level causing ionic imbalance, osmotic stress, excessive reactive oxygen species (ROS), and early senescence [1,2]. On the other hand, phosphorus (P) deficiency hampers the photosynthesis system and produces ROS as well [3,4]. ROS are cytotoxic, causing damage cellular organelles like proteins, DNA, lipids, and carbohydrates through causing an imbalance in their neutralizing system. Under both salinity and low P stresses, ROS, like superoxide radicals (O_2_^•−^), hydroxyl radical (OH^•^), hydrogen peroxide (H_2_O_2_), and singlet oxygen (^1^O_2_), are produced in different cellular compartments [5]. Like ROS, methylglyoxal (MG) is highly mutagenic and cytotoxic that accumulates in plant cells under various abiotic stress like salinity [6,7,8]. However, MG production under low P has not been yet reported. Higher accumulation of MG in cells also interacts with different cellular organelles such as DNA, proteins, and lipids [9]. At the same time, increased MG can promote ROS generation by altering and deactivating the antioxidant system [8] or by hampering of photosynthesis system [10]. Therefore, both ROS and MG must be maintained under a sub-lethal level for cellular survival.

Plants have their internal antioxidant defense system comprising of both enzymatic and nonenzymatic components to protect themselves against oxidative damages [11]. Enzymatic components are superoxide dismutase (SOD), peroxidase (POD), catalase (CAT), ascorbate peroxidase (APX), glutathione peroxidase (GPX), monodehydroascorbate reductase (MDHAR), dehydroascorbate reductase (DHAR), and glutathione reductase (GR) whereas ascorbic acid (ASA) and glutathione (GSH), α-tocopherol, and carotenoids are non-enzymatic antioxidants [5]. In contrast, detoxification of MG occurs by glyoxalase-I (Gly-I) and glyoxalase-II (Gly-II). MG metabolism is catalyzed by Gly-I in the presence of GSH to form an intermediate compound *S*-lactoylglutathione (SLG), which is further modified to lactic acid in the presence of Gly-II [9]. A good number of researchers have reported that increased tolerant of plants correlates with the induction of the antioxidative and glyoxalase systems under salinity, drought, and other stresses [7,12,13,14].

Salt tolerance is a multigenic controlled complex trait to its adaptive responses; it is involved with a complex mechanism involving biochemical, physiological, and molecular approaches [15]. Salinity causes osmotic stress, and osmotic management is also a very important mechanism for salt tolerance [16]. Trehalose (Tre), a non-reducing disaccharide, is one of the important osmoprotectants for osmotic adjustment in plants under stress [17,18]. It is not only an energy source under desiccation, but an efficient stabilizer for dehydrated enzymes, proteins, and lipid membranes, as well as for other biological structures [19]. It is also a signaling and antioxidant molecule that acts as an activator of genes involving stress response and detoxification. Unfortunately, Tre synthesis in the plant is insufficient to protect against adverse effects under stress. Consequently, exogenous Tre has been reported to increase salinity tolerance by modulating antioxidants [20,21,22]. Improved resistance to high salinity with osmotic adjustment by Tre application was also reported in *Catharanthus roseus* and Arabidopsis with ionic regulation [23,24]. Moreover, MG detoxification systems by glyoxalase under low P has not been found in any report. At the same time, the response of Tre in enhancing tolerance under oxidative stress under P starvation condition has not yet been reported.

Maize is a P-loving crop and produces low P symptoms. As an important protectant, it may play an important role in oxidative stress under salinity and low P stress in maize. Considering those points, we designed a study in a hydroponic system to examine the role of exogenous Tre in the maintenance of saline and low P mediated growth, oxidative stress, lipid peroxidation, ion homeostasis, antioxidant responses, and MG detoxification.

## 2. Results

Analysis of variance (ANOVA) indicated that treatment variances for all the traits studied were statistically significant (Table 1). On the other hand, for variety, a significant difference was found in Na^+^/K^+^, O_2_^•−^, H_2_O_2_, MDA, LOX, SOD, CAT, APX, GR, MDHAR, GST, and Gly-I. In contrast, the interaction was significant for root volume, Tre, MDA, POD, APX, GR, MDHAR, DHAR, and Gly-I. The significant differences among treatment for all the traits suggest that the application of Tre may have an important role in the growth and biochemical parameters related to oxidative stress under saline and low P stress.

**Figure 1:**
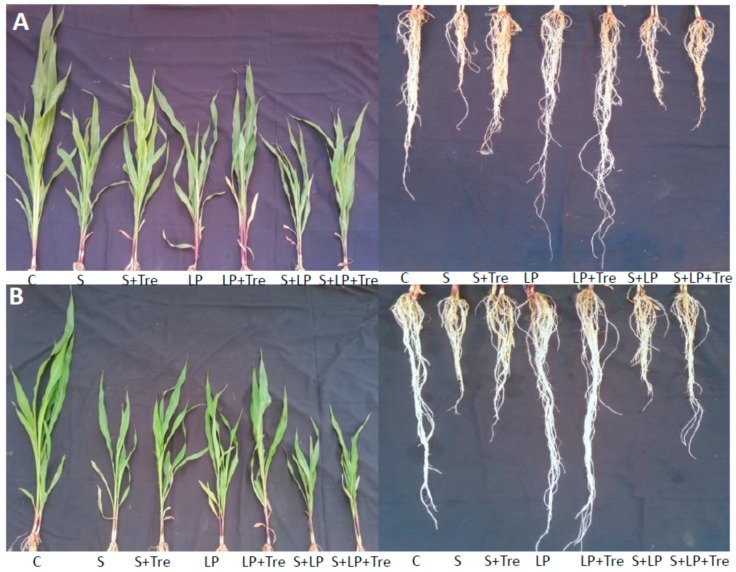
Effect of Tre on the shoot and root lengths (cm) of maize under saline (S), low P (LP), and combined (S + LP) stress in BHM-7 (**A**) and BHM-9 (**B**).

A significant variation was found among the treatments for all growth parameters (Table 1, Table 2). However, the interaction effect was significant for root volume only. Data showed that saline, low P, as well as their combined stress, reduced the shoot and root length, and root volume as compared to the respective control, and the reduction was significant in saline and combined stress (Table 2, Figure 1). Importantly, the application of Tre increased the shoot length in all the stresses (Table 2, Figure 1). In contrast, Tre increased root volume in low P and combined stress. However, the increased root volume in saline stress was not statistically significant. For a variety effect, significant variation was not found. Tre application significantly increased root volume in saline stressed seedlings of BHM-7, although the increment of root volume in BHM-9 was not statistically significant. It also implicated that increments of root volume in combined stressed seedlings by Tre were significant compared to that in stressed seedlings without Tre in both varieties.

Both treatment and varietal effects were significant for Na^+^/K^+^ (Table 1 and Table 3). Data showed that saline, low P, and combined stress either increase Na^+^ uptake and/or efflux of cellular K^+^. Tre application decreased the values of Na^+^/K^+^ significantly in saline and combined stress compared to the stresses without Tre. However, Tre decreased Na^+^/K^+^ slightly (9%) in low P stress. For P content, the only treatment effect was significant, where all the stresses decreased the P content significantly compared to that in control (Table 3). Tre application increased the P content significantly in saline, and low P stressed seedlings. Although Tre increased the P content in the seedlings of combined stress by 21% and it removed P deficiency symptoms in maize leaves in both maize varieties (Table 3; Figure S1). Both treatment and interaction effects were significant for Tre content (Table 3), which was attributed due to significant reduction of cellular Tre under all the stresses in both varieties as compared to non-stress control. The interaction effect also showed that the application of exogenous Tre in all the stress treatments improved Tre concentration in leaves significantly in both varieties compared to the stresses without Tre (Table 3).

Treatment and variety effects were significant for ROS (O_2_^•−^ generation rate and H_2_O_2_ concentration) production (Table 4). All the stress treatments increased the ROS enormously, which were statistically significant compared to non-stress control. The content of O_2_^•−^ was 130%, 70%, and 148% higher in salinity, low P, and combined stress, respectively, over control while H_2_O_2_ content was 99%, 99%, and 160% higher in salinity, low P, and combined stress, respectively. It was notable that Tre application in stress treatments caused a significant reduction in O_2_^•−^ and H_2_O_2_ production over stress treatments without Tre (Table 4). In the case of MDA content, treatment, variety and interaction effect were significant (Table 1 and Table 4). In interaction effect, as compared to control, 109%, 159%, and 181% higher concentration of MDA were observed in saline, low P, and combined stress, respectively, in BHM-7. On the other hand, the concentration was 51%, 58%, and 152% higher in BHM-9 under saline, low P, and combined stress, respectively. Tre reduced the MDA content significantly in all the stress treatments (Table 4). In contrast, treatment and variety effects were significantly independent for LOX activity (Table 1 and Table 4). Like ROS and MDA, LOX activity also increased enormously and significantly in comparison to control in all the stress treatments (Table 4). Compare to control, saline, low P, and combined stress increased the activity by 133%, 157%, and 254%, respectively. It was remarkable that Tre application decreased the LOX activity significantly in all the stress treatments (Table 4).

Superoxide dismutase activity differed significantly among the treatments and varieties (Table 5). The activity was significantly higher in saline and combined stress as compared to control, but the increased activity in low P stress was statistically similar to control. Tre increased the SOD activity significantly in combined stress (Table 5). Tre also increased the activity in saline and low P stress, but they were statistically similar to the activity in these stresses. Among the varieties, BHM-7 contained significantly higher activity than BHM-9 (Table 5). Unlike SOD activity, the interaction effect was significant for POD activity (Table 5). As compared to control, all the stresses increased the activity in BHM-7, although they were statistically similar. On the other hand, saline and low P stress increased the activity significantly in BHM-9. In BHM-7, Tre decreased the activity in all the stresses in both varieties. In the case of CAT activity, variety and treatment effects were significant individually (Table 5). All of the stresses increased CAT activity compared to that in control, although they were statistically similar. Application of Tre enhanced the activity in all the stresses, and in combined stress, the activity was significantly higher than the same stress without Tre. Treatment and interaction were significant for GPX activity, whereas treatment and variety were significant individually and jointly for APX activity (Table 5).

All the stress significantly changed the GPX activity compare to control in BHM-7, being significantly higher in saline and low P stress while it was significantly lower in combined stress. On the other hand, in BHM-9, significantly increased activity was observed in saline stress only. Low P also increased the activity insignificantly, but combined stress decreased the activity. Interestingly, Tre improved the activity significantly in saline and combined stress in both varieties. Tre also increased the activity in low P stress by 16% in BHM-9 only. Data of interaction effect for APX revealed the activity was decreased significantly in both varieties by all the stresses (Table 5). However, Tre application did not increase the activity in stress treatments in both varieties. GR activity was significant for treatment and variety individually (Table 1 and Table 5).

As compared to control, significantly higher GR activity was found in saline and combined stress, although low P induced the activity to some extent. The presence of Tre in saline and combined stress induced the activity substantially than the same treatments without Tre. However, BHM-7 maintains higher GR activity than BHM-9 (Table 5). A significant interaction effect of MDHAR activity implicated notable increases in the activity under all stresses in both varieties (Table 5). However, Tre application was successful in increasing the activity in both varieties in all stresses. The interaction effect was also significant for DHAR activity (Table 1 and Table 5). Under salinity, BHM-7 maintained almost similar activity, although the activity considerably decreased under low P and combined stress. In BHM-7, Tre restored the activity by 11%, 32%, and 20% in saline, low P, and combined stress, respectively. Correspondingly, Tre restored the activity in BHM-9 by 13%, 39%, and 46%, respectively. Glutathione *S*-transferase (GST) activity was significant for treatment and variety (Table 1 and Table 5). All the stresses induced the activity, where the increases were statistically significant in saline and low P stress compared to control, but in combined stress, the activity was only 13% higher. Tre caused a significant improvement in GST activity in saline stress. However, the increased activity by Tre in low P and combined stress was statistically similar to the stress without Tre. Importantly, BHM-7 possessed significantly higher GST activity than BHM-9 (Table 5).

Treatment effect individually affected MG content significantly in leaves of maize seedlings (Table 1 and Table 6). Saline, low P, and combined stress strongly enhanced the content of MG which was 2.1-, 2.35-, and 2.95-fold in magnitude over control. The application of Tre significantly decreased the MG content in stress treatments. Treatment and variety, either individually or interactively, changed Gly-I activity in leaves of maize seedlings. Tre increased the activity in low P stress significantly in both varieties. However, in other stresses, the activity either remained similar or decreased. Notably, BHM-9 maintained higher Gly-I activity under low P and combined stress (Table 6). The only treatment effect was significant for Gly-II activity (Table 1 and Table 6). As compared to control, all the stresses reduced Gly-II activity. Importantly, the activity was significantly enhanced by Tre in all the stress treatments. However, the activity did not differ significantly in the varieties (Table 6).

## 3. Discussion

Under stress conditions, many plants produce compatible solutes or osmoprotectants to protect against osmotic stress [16,25]. In this study, we studied the possible governing role of exogenous Tre application on ROS and MG metabolism under saline, low P, and their combined stress in maize seedlings.

Salinity and combined stress reduced the length of shoot and root as well as root volume in both genotypes. However, in low P stress, the root length and volume increased. Tre enhanced the growth of shoots and roots in both maize genotypes in all stresses. This salinity mediated decrease in shoot and root data might be due to the alteration of metabolic activities affected by the increase Na^+^ as we found higher Na^+^/K^+^ in leaves of both genotypes. The higher concentration of Na^+^ in soil decreases water uptake and increases the accumulation of Na^+^ in cells resulting in osmotic stress and alteration of cellular metabolism [7,20]. Therefore, ion toxicity and osmotic stress can imbalance the growth of root and shoot in both genotypes. Tre has been reported to improve leaf water in plants under salinity and drought in rice [20,21,22]. Therefore, improved growth parameters by Tre application might also be through improving leaf water resulting in lower concentrations of Na^+^ under saline and combined stress in maize [26,27]. The application of Tre was effective in promoting growth parameters than those of the stressed plants without Tre. Tre may reduce cell damage under saline added treatments as it decreases the accumulation of Na^+^ in salinity in these treatments. The beneficial effect of Tre has also been reported in rice [21,22]. However, a beneficial role in maize by Tre under low P is not clear. Increased P in maize leaves, as well as lessened P deficiency symptoms suggest that Tre can help in P uptake in maize. Previously, increased root systems in Pi-starved plants by exogenous application of sucrose was reported by Jain et al. [28]. Pi-starvation responses in plants by carbohydrate signals were also reported by many researchers [26,29,30,31,32].

Salinity is the most disturbing abiotic stress in plants with a negative consequence on gas exchange, resulting in low CO_2_ assimilation for photosynthesis, and subsequently, significant reduction of electron transportation resulting in higher ROS production [5,33,34]. In both cases, ROS are produced, which are highly cytotoxic to cell organelles like DNA, protein, lipid, and pigment [5]. In this study, significantly higher ROS (O_2_^•−^, H_2_O_2_) were found in seedlings of saline, low, and combined stress in contrast to those in control treatment, which is an indicator of oxidative bursts in the leaf tissues of maize. Increased ROS cause lipid peroxidation, a potential biomarker of oxidative damage to membranes, causing electrolyte leakage, loss of membrane permeability, and malfunctioning of membrane proteins and ion channels [1,35,36]. In our results, the higher ROS produced higher MDA (lipid peroxidation product) along with its related enzyme, LOX. Therefore, higher ROS and MDA can damage root and shoot tissue in maize seedlings. At the same time, the higher concentration of potential cytotoxic MG can damage the growth of maize seedlings. Salinity mediated oxidative damage in maize was also reported in our previous study [7]. However, low P mediated higher ROS and MDA are limited in maize, and the regulation of MG in low P condition has not yet been reported. Zhang et al. [3] reported significantly higher O_2_^•−^, H_2_O_2_, and MDA as compared to control in maize leaves under low P stress. ROS production under low P was also reported in mulberry [37], rice [4], and bean [38]. Exogenous Tre was also reported to alleviate ionic unbalance and ROS bursts under salinity in *Arabidopsis* seedlings [24].

Under abiotic stress, including salinity, plants accumulate sugars and other compatible solutes which serve as osmoprotectants and in some cases, stabilize biomolecules under stress conditions [39]. Tre is reported to have an important physiological role as an abiotic stress protectant through its ability to scavenge ROS, conferring protection to the machinery of protein synthesis [40,41]. In this study, the application of exogenous Tre improved the concentration of Tre in leaves. Consequently, it decreased the accumulation of Na^+^, resulting in higher K^+^/Na^+^ ration. Contrary, the accumulation of P in the presence of Tre is not clear. One of the causes might be due to higher intake by increased root length and volume.

Application of Tre decreased the salinity- and low P-mediated higher concentrations of O_2_^•−^, H_2_O_2_, MDA, MG, as well as LOX activity in all the stress treatments. Production of MDA in all the stresses was directly related to ROS and LOX activity. This is in agreement with several studies on salinity and low P stress [3,7,22,34]. This relation can be explained by considering antioxidant and glyoxalase activities. Besides, Tre mediated inhibition of LOX activity is likely to lessen MDA production in the maize leaf. Upon Tre application, decreased MDA contents under salinity were also reported in rice [22]. Importantly, in this study, Tre decreased the MDA production in maize under low P and combined stress.

In plant cells, SOD is considered to provide primary protection against O_2_^•−^, which is converted to H_2_O_2_ for subsequent metabolism by CAT and peroxidase enzymes. In the present study, SOD activity increased in saline, low P, and combined stressed seedlings than that in control seedlings. Increased SOD activity along with higher O_2_^•−^ generation rates suggest a relation between increased production of ROS and a protective mechanism to lessen the oxidative damage caused by the stresses. SOD activity increment in salinity stress was also noticed in different crops including maize [7,21,42]. However, SOD activity regulation by low P has not yet been found in maize. On the other hand, low P mediated increased SOD that was reported in rice [4] and mulberry [37]. Contrary, Juszczuk et al. [38] reported that P deficiency did not affect SOD activity in bean (*Phaseolus vulgaris* L.). Therefore, SOD activity under low P depends on plant species. Application of Tre in stress treatments increased SOD activity remarkably compared to stress treatments without Tre in both genotypes. The reduction of O_2_^•−^ along with enhanced SOD activity in the presence of Tre suggest its role in ROS metabolism. This result is in good agreement with Shahbaz et al. [22], where Tre as a foliar spray upregulated the activity in rice. Lower regulation of O_2_^•−^ activity by Tre was also reported in rice [20,21,41]. On the other hand, the deceased O_2_^•−^, along with higher SOD activity by Tre in low P stress in both maize seedlings, suggests its O_2_^•−^ scavenging role in the plant under P starvation.

In plants, H_2_O_2_ is scavenged by CAT, POD, GPX, and APX into water [43,44]. Compare to APX, GPX, and POD, CAT shows low affinity to H_2_O_2_ with a high processing rate. Therefore, it can play a principal role in H_2_O_2_ scavenging in plants under salinity stress. It is true because CAT is independent of other cellular reductants for instituting its activity [45]. However, in our previous study, CAT was genotype-dependent in maize, and increased activity was found in a drought tolerant genotypes [7]. In this study, genotype BHM-9 had comparatively higher CAT activity in both salinity and low P stresses, suggested its higher H_2_O_2_ metabolizing capacity. The beneficial effects of Tre stimulated the CAT activities in saline, low P and combined stresses. Peroxidase (POD), a heme-containing enzyme, is important for quencher of reactive intermediary forms of O_2_ and peroxy radicals under stressed conditions [46]. In this study, POD activity was induced significantly by salinity and low P stress in both genotypes, being statistically similar in combined stress when compared to that in control. Therefore, POD plays important role ROS metabolism and thus, conferred tolerant to maize seedlings under all the stresses. The induced activity of POD under salinity was reported in soybean [47], liquorice [48], and in *Lepidium sativum* [49], whereas drought stress-induced POD activity was also reported in rapeseed [50], rice [20,21,22], and liquorice [48]. On the other hand, enhanced POD activity under low P was also reported in rice [4]. In this study, Tre application in all the stress treatments inhibited the activity. Decreased activity by Tre application in salinity treated rice was also reported by Shahbaz et al. [22]. However, Tre mediated POD activity in low P stressed in maize or other plants have not yet been reported.

Glutathione peroxidases (GPXs), a large and diverse isozyme family, use GSH to reduce H_2_O_2_ and organic and lipid hydroperoxides to escape oxidative [51] stress. GPXs were also reported to be involved in many studies demonstrating the significant stress mitigating role under stress [7,52,53,54,55]. However, higher GPX activity in low P stress has not yet been reported. In our study, higher GPX activity under salinity and low P stress can play an important role to reduced oxidative stress in maize. At the same time, the enhancement of GPX activity in Tre application along with lower ROS suggests its beneficial role in H_2_O_2_ detoxification in maize in all stress treatments.

The ascorbate-glutathione cycle is reported as the most important anti-oxidation metabolic pathway that participates in H_2_O_2_ metabolism and redox maintenance [44,56]. Enzyme like APX, GR, MDHAR, and DHAR are very important in this cycle, where APX reduces H_2_O_2_ to H_2_O through oxidation of ASA to protect plants from oxidative damage under abiotic stress. In our study, increased APX activity in saline and low P stress indicated its ROS detoxification role. On the other hand, combined stress inhibited the activity. Importantly, Tre application in all the stresses enhanced APX activity, which improved tolerance in maize seedlings of both genotypes through H_2_O_2_ metabolism. GR is essential to recycle GSH in the ascorbate-glutathione cycle in an NADPH-dependent reaction. In the present study, enhanced APX activity by salinity and low P can diminish H_2_O_2_ mediated oxidative damage. However, in combined stress, the activity was inhibited. Importantly, the APX activity was further enhanced in the application of Tre in all the stresses. Higher APX activity by Tre was also reported in rice under salinity by several research groups [20,57]. On the other hand, enhanced APX activity in low P stressed seedlings is in good agreement with Zhang et al. [3], where they found a remarkable increase in APX activity along with ASA in P starved maize leaves. However, further enhancement of the activity in maize leaves by Tre in this study indicated a beneficial role of Tre to detoxify H_2_O_2_. The Tre mediated enhanced activities of MDHAR and DHAR in saline- and low P- and both treated seedlings suggest that Tre has an important role in AsA maintenance in maize seedlings. On the other hand, the change in GR activity differed with maize genotypes, being higher in BHM-9 under a single treatment. However, the activity was strongly inhibited in combined stressed seedlings. Application of Tre enhanced GR activity in all the stress treatments suggesting its influence in GSH maintenance and, thus, conferred tolerance in maize genotypes under the stress conditions.

In this study, the higher GST activity with or without Tre in saline, low P, and combined stress can play an important role in hydroperoxide metabolism and/or leaf senescence. Zhang et al. [3] reported that increased activity of GST in maize leaf under low P is likely to protect maize from damage caused by abiotic stresses through scavenging ROS. However, to our best knowledge, this is the first report to change GST activity by Tre in low P stress in the plant [58].

MG is unavoidably produced enzymatically or spontaneously; however, under abiotic stress, its overproduction is cytotoxic to the cellular organelle [5,8,9]. It is also reported that MG is related to O_2_^•−^ generation [10], and ROS and MG detoxification is a coordinated effect of antioxidants and glyoxalases [13,14,59,60]. The result of the study showed that Gly-I and Gly-II activities were enhanced under salinity and varied with genotypes in maize, being upregulated in BHM-9. In our previous study, we found higher Gly-I and Gly-II activities in inbred saline tolerant maize [7]. Higher glyoxalase activities also reported in rice under salinity [14]. Therefore, higher glyoxalases in BMH-9 under salinity suggested better MG detoxification. However, Low P and combined stress downregulated the activities. Importantly, Tre application increased the activities of Gly-I and Gly-II in both genotypes under all stresses. The upregulation of glyoxalase activities under salinity by Tre has been reported in other crops. However, their activities under low P is not available. Therefore, it is possible that Tre can play an important role in MG detoxification in maize under P starvation conditions.

## 4. Materials and Methods

### 4.1. Plant Materials and Stress Treatments

Seedlings of two maize genotypes BARI hybrid Maize-7 (BHM-7) and BARI hybrid Maize-9 (BHM-9) were grown in rock medium. Seedlings of five-day-old were transferred on a hydroponic system containing Hoagland nutrient solution supplied with 1000 µM KH_2_PO_4_. After three days hardening, the seedlings were transferred to seven treatments: Control (only Hoagland solution supplied with 1000 µM KH_2_PO_4_), salinity (150 mM salinity induced by NaCl), salinity + Tre (Tre, 10 mM), low P (Hoagland solution supplied with 5 µM KH_2_PO_4_), salinity + Tre, and combined stress (salinity + low P + Tre). The concentration of Tre was chosen from previous studies [24]. The pH of the nutrition solution was maintained at 6.0, and the nutrient solution was replaced every 3 days. The air was supplied to avoid hypoxia conditions. The experimental temperature was 25–28/18–20 °C (day/night) with a 14 h light cycle (600–700 µmol m^−2^ s^−1^) and relative humidity approximately 60–65%. The experiments were repeated three times and data were taken on different parameters from uppermost fully expanded leaves after fifteen days.

### 4.2. Growth Parameter Measurement

Shoot length, root length, and volume were measured by a scale and measuring cylinders.

### 4.3. Measurement of Na^+^/K^+^

The leaf extract was placed on compact Na^+^ ion meter (Horiba-731, Japan) and compact K^+^ ion meter (Horiba-722, Tokyo, Japan) to measure Na^+^ and K^+^ ions. The leaves were washed three times with deionized water and wiped properly. A tissue sap extractor (provided by Horiba, Tokyo, Japan) was used to extract leaf saps. The sap was directly applied to the ion meters to measure the readings of Na^+^ and K^+^ in ppm. The ration of Na^+^ to K^+^ was calculated from the estimated values.

### 4.4. Determination of P Concentration in Leaf

The fully expanded uppermost leaves were subjected to dry at 80 °C for 48 h. The content of elemental P was determined colorimetrically following the molybdate blue ascorbic acid method of Olsen and Sommers [61].

### 4.5. Estimation of Tre Content

Tre content was determined spectrophotometrically following the method of Bhuiyan et al. [62]. Data of absorbance were noted at 630 nm, and Tre concentration was calculated as micromoles per gram fresh weight (µmol g^−1^ FW) comparing with a standard curve of commercial Tre (Sigma-Aldrich, USA).

### 4.6. Measurement of the O_2_^•−^ Generation Rate and H_2_O_2_

Generation of O_2_^•−^ was determined as per Elstner and Heupel [63]. The O_2_^•−^ generation was measured by comparing a standard curve of NaNO_2_. H_2_O_2_ was assayed according to the method of Yu et al. [64], and calculated with an extinction coefficient of 0.28 μM^−1^ cm^−1^.

### 4.7. Measurement of MDA

The content of MDA (as lipid peroxidation) was according to Heath and Packer [65]. MDA was calculated with an extinction coefficient of 155 mM^−1^ cm^−1^ and expressed as nmol g^−1^ FW.

### 4.8. Measurement of MG

The content of MG in leaf tissue was measured by using N-Acetyl cysteine, according to the description in Rohman et al. [7].

### 4.9. Extraction of Enzyme Solution and Assay Activity

The soluble enzyme was extracted from maize leaf following the method of Rohman et al. [7]. Fresh leaf tissue (0.5g) was homogenized in 1 mL of 50 mM ice-cold potassium phosphate (K-P) buffer (pH 7.0) by mortar and pestle. The buffer contained 100 mM KCl, 1 mM ascorbate, 5 mM β-mercaptoethanol, and 10% (*w/v*) glycerol. The homogenates were centrifuged at 11,500× *g* for 10 min, and the supernatants were used as enzyme solution to assay activities. All procedures were performed below 4 °C.

### 4.10. Determination of Protein

The protein was quantified following Bradford [66].

### 4.11. Assay of Enzymatic Activities

The method of Doderer et al. [67] was carried out to assay LOX (EC: 1.13.11.12) activity from the soluble enzyme solution. The activity was written as μmol min^−1^ mg^−1^ protein considering the extinction coefficient of 25,000 M^−1^ cm^−1^. SOD (EC 1.15.1.1): SOD activity was assayed based on the competition between SOD and NBT for the production of superoxide from xanthine and xanthine oxidase interaction following Spitz and Oberley [68]. One unit of activity was defined as the protein amount needed to inhibit 50% inhibit of NBT. POD (EC 1.11.1.7): POD activity was assayed following Hemeda and Klein [69] by guaiacol oxidation for 1 min and the activity was calculated with an extinction coefficient of 26.6 mM^−1^ cm^−1^. CAT (EC: 1.11.1.6): CAT activity was assayed with the method described in Bhuiyan et al. [62]. The activity was calculated with an extinction coefficient of 39.4 M^−1^ cm^−1^ H_2_O_2_ decompositions. GPX (EC: 1.11.1.9): GPX activity was assayed following the description of Rohman et al. [7], where an extinction coefficient of 6.62 mM^−1^ cm^−1^ was used to calculate the activity. The activity of APX (EC: 1.11.1.11) was assayed according to the process of Nakano and Asada [70]. The activity was calculated considering an extinction coefficient of 2.8 mM^−1^ cm^−1^. The activities of MDHAR (EC: 1.6.5.4) and GR (EC: 1.6.4.2) were assayed following the methods of Bhuiyan et al. [62] and were calculated using extinction coefficients of 6.2 mM^−1^ cm^−1^. On the other hand, DHAR (EC: 1.8.5.1) activity was determined with the procedure of Nakano and Asada [70], where an extinction coefficient of 14 mM^−1^ cm^−1^ was used to calculate the activity. GST (EC: 2.5.1.18) activity was determined using model substrate 1-chloro-2,4-dinitrobenzene following the method used by Rohman et al. [7]. The activity was calculated using an extinction coefficient of 9.6 mM^−1^ cm^−1^. The activities of Gly-I (EC: 4.4.1.5) and Gly-II (EC 3.1.2.6) were assayed following the methods of Yadav et al. [6] and Bhuiyan et al. [62], respectively. The activities were calculated using extinction coefficients of 3.37 and 13.6 mM^−1^ cm^−1^, respectively.

### 4.12. Statistical Analysis

The experiments were carried out following a completely randomized design (CRD) with three replications, and each experiment was repeated three times. Analysis of variance was performed following factorial CRD design, where the individual effect of treatment and variety and their interaction were observed. Data obtained from the experiments were analyzed by statistical software Statistix-10. The means of treatment, variety, and interaction were compared using the least significant difference (LSD) test at a significant level of *p* ≤ 0.05. In the case of non-significant interaction, the mean separation was not performed with lettering.

## 5. Conclusions

The above data show that salinity, low P, and their combined stress hamper the growth of maize and hinder P and Tre intake, being severe in combined stress treatment. The production of ROS in both genotypes is associated with membrane damage. The changes in antioxidative enzymes under the stresses suggest that they intensify the oxygen-scavenging system to remove the ROS and to maintain the balance of ROS for defending seedlings against oxidative damage, although the regulation of the enzymes differed between salinity and low P stresses in many cases. Greater oxidative damage under combined stress results from the inhibition of most of the enzymes. Glyoxalases activities differ with genotypes and treatments where the intensified activities are observed in BHM-9 under salinity only. However, comparative lower ROS, MDA contents, and LOX activity along with higher ROS scavenging enzyme activities suggest that BHM-7 might have better tolerance to salinity stress. The application of Tre in the stress treatments improves the growth of shoots and roots as well as lessens the oxidative damage by maintaining ROS, lipid peroxidation, and MG by enhancing enzymatic antioxidants and glyoxalase, although GR activity is not enhanced in low P stress which can be a threat in the recycling of the GSH pool to maintain GSH dependent ROS scavenging as well as MG detoxifying reactions. Taken together, we find the important role of Tre application in the study period under salinity and low P stress. However, how plants manage ROS though antioxidants and glyoxalases in the long-term, particularly low P and low P plus salinity stresses, warrants more research.

## Figures and Tables

**Table 1:** Analysis of variance for shoot length (cm), root length (cm), root volume (cm^3^), Na^+^/K^+^, P (%), Tre (µmol g^−1^ FW), O_2_^•−^ (nmol g^−1^ FW min^−1^, H_2_O_2_ (µmol g^−1^ FW), malondialdehyde (MDA; nmol g^−1^ FW), activities of lipoxygenase (LOX; µmol min^−1^ mg^−1^ protein), superoxide dismutase (SOD; unit min^−1^ mg^−1^ protein), peroxidase (POD; µmol min^t1^ mg^−1^ protein), catalase (CAT; µmol min^−1^ mg^−1^ protein), glutathione peroxidase (GPX; nmol min^−1^ mg^−1^ protein), ascorbate peroxidase (APX; µmol min^−1^ mg^−1^ protein), glutathione peroxidase (GR; nmol min^−1^ mg^−1^ protein), monodehydroascorbate reductase (MDHAR; nmol min^−1^ mg^−1^ protein), dehydroascorbate reductase (DHAR; nmol min^−1^ mg^−1^ protein), GST (nmol min^−1^ mg^−1^ protein), methylglyoxal (MG; µmol g^−1^ FW), Gly-I (µmol min^−1^ mg^−1^ protein) and Gly-II (µmol min^−1^ mg^−1^ protein) of maize seedlings under salinity and low P stresses influenced by exogenous trehalose (Tre, 10 mM). *, ** and ^NS^ mean significant at *p* ≤ 0.05, *p* ≤ 0.01, and non-significant, respectively.

Source of Variation	d.f.	Mean Sum of Square										
		Shoot Length	Root Length	Root Volume	Na^+^/K^+^	P	Tre	O_2_^•−^	H_2_O_2_	MDA	LOX	SOD
Treatment	6	2047.2 **	2534.7 **	191.3 **	0.965 **	0.397 **	5.55 **	47.3 **	190.8 **	2988.7 **	1964.5 **	4058.9 **
Variety	1	148.2 ^NS^	369.9 ^NS^	0.647 ^NS^	0.264 **	0.001 ^NS^	0.216 ^NS^	6.72 *	23.7 **	9711.2 **	492.1 **	1571.1 *
Treatment × Variety	6	65.9 ^NS^	44.0 ^NS^	6.92 **	0.030 ^NS^	0.015 ^NS^	0.239 *	2.24 ^NS^	4.27 ^NS^	341.7 **	71.6 ^NS^	91.4 ^NS^
Error	112	103.4	100.5	1.47	0.018	0.022	0.107	1.09	3.06	82.51	43.45	260.3
		Mean sum of square										
		POD	CAT	GPX	APX	GR	MDHAR	DHAR	GST	MG	Gly-I	Gly-II
Treatment	6	0.274 **	368.3 **	5889.7 **	0.599 **	2298.5 **	381.2 **	16,881.7 **	11,203.5 **	320.9 **	0.219 **	0.005 **
Variety	1	0.0127 ^NS^	1532.2 **	38.2 ^NS^	0.917 **	1495.7 **	3059.6 **	15.8 ^NS^	3296.4 *	0.001 ^NS^	0.917 **	0.0001 ^NS^
Treatment × Variety	6	0.030 *	41.4 ^NS^	797.4 ^NS^	0.088 **	91.5 **	166.8 *	2073.5 *	265.5 ^NS^	0.001 ^NS^	0.111 **	0.0004 ^NS^
Error	112	0.01	54.1	366.5	0.03	79.1	59.6	710.7	618.6	7.04	0.015	0.0002

**Table 2:** Effect of Tre on shoot length, root length and root volume in maize seedlings under saline and low P stress. Each value of data represents mean of three independent experiments, each replicated three times. Values within a column with different letters are significant at *p* ≤ 0.05 applying least significant difference (LSD) test.

Source of Variation	Shoot Length (cm)	Root Length (cm)	Root Volume (cm^3^)
Treatment			
Control (C)	67.8 ^a^	50.9 ^a^	10.7 ^b^
Saline (S)	41.9 ^ef^	33.8 ^bc^	5.67 ^c^
S + Tre	51.7 ^cd^	38.8 ^b^	6.46 ^c^
Low P (LP)	52.3 ^c^	55.0 ^a^	11.3 ^b^
LP + Tre	60.3 ^b^	57.2 ^a^	12.12 ^a^
S + LP	37.1 ^f^	28.5 ^c^	3.84 ^d^
S + LP + Tre	45.0 ^de^	31.8 ^c^	6.05 ^c^
SE	3.39	3.34	0.40
*F ratio (df = 6, 112)*	19.8	25.2	130.3
Variety			
BHM-7	49.8 ^a^	40.6 ^a^	7.94 ^a^
BHM-9	51.9 ^a^	44.0 ^a^	8.09 ^a^
SE	1.81	1.79	0.22
*F ratio (df = 1, 112)*	1.43	3.68	0.44
Interaction			
C × BHM-7	65.4	46.4	10.4 ^c^
C × BHM-9	70.2	55.4	11.0 ^bc^
S × BHM-7	38.5	34.2	4.34 ^f^
S × BHM-9	45.3	33.3	7.00 ^d^
S + Tre × BHM-7	49.7	38.3	6.50 ^de^
S + Tre × BHM-9	53.8	39.2	6.41 ^de^
LP × BHM-7	52.5	53.3	11.2 ^a-c^
LP × BHM-9	52.2	56.8	11.3 ^a-c^
LP + Tre × BHM-7	61.3	55.7	12.3 ^a^
LP + Tre × BHM-9	59.3	58.8	11.90 ^ab^
S + LP × BHM-7	34.8	26.2	4.20 ^f^
S + LP × BHM-9	39.5	30.9	3.49 ^f^
S + LP + Tre × BHM-7	46.5	29.9	6.60 ^de^
S + LP + Tre × BHM-9	43.5	33.7	5.50 ^e^
SE	4.79	4.73	0.57
*F ratio (df = 6, 112)*	0.63	0.43	4.71

**Table 3:** Effect of Tre on the ratio of sodium and potassium ion (Na^+^/K^+^), P, and Tre accumulation in leaves of maize seedlings under saline and low P stress. Each value of data represents the mean of three independent experiments, each replicated three times. Values within a column with different letters are significant at *p* ≤ 0.05 applying the LSD test.

Source of Variation	Na^+^/K^+^	P (%)	Tre (µM g^−1^ FW)
Treatment			
Control (C)	0.39 ^d^	0.79 ^a^	2.35 ^b^
Saline (S)	0.67 ^b^	0.55 ^c^	1.70 ^d^
S + Tre	0.57 ^c^	0.72 ^ab^	2.69 ^a^
Low P (LP)	0.41 ^d^	0.54 ^c^	1.61 ^de^
LP + Tre	0.37 ^d^	0.69 ^b^	2.06 ^c^
S + LP	1.00 ^a^	0.38 ^d^	1.08 ^f^
S + LP + Tre	0.75 ^b^	0.46 ^cd^	1.43 ^e^
SE	0.05	0.05	0.11
*F ratio (df = 6, 112)*	43.4	21.7	52.0
Variety	0.64 ^a^	0.59 ^a^	1.81 ^a^
BHM-7	0.55 ^b^	0.58 ^a^	1.88 ^a^
BHM-9	0.27	0.02	0.06
SE	11.9	0.001	2.03
*F ratio (df = 1, 112)*			
Interaction			
C × BHM-7	0.37	0.75	2.35 ^bc^
C × BHM-9	0.41	0.83	2.34 ^bc^
S × BHM-7	0.75	0.51	1.75 ^ef^
S × BHM-9	0.59	0.59	1.65 ^fg^
S + Tre × BHM-7	0.59	0.73	2.45 ^b^
S + Tre × BHM-9	0.55	0.71	2.94 ^a^
LP × BHM-7	0.44	0.56	1.48 ^fg^
LP × BHM-9	0.38	0.51	1.74 ^ef^
LP + Tre × BHM-7	0.4	0.68	1.97 ^de^
LP + Tre × BHM-9	0.34	0.69	2.14 ^cd^
S + LP × BHM-7	1.1	0.41	1.14 ^hi^
S + LP × BHM-9	0.9	0.35	1.02 ^i^
S + LP + Tre × BHM-7	0.83	0.48	1.49 ^fg^
S + LP + Tre × BHM-9	0.68	0.43	1.38 ^gh^
SE	0.08	0.06	0.15
*F ratio (df = 6, 112)*	1.36	0.83	2.24

**Table 4:** Effect of Tre on O_2_^•−^ generation rate, H_2_O_2_, MDA, and LOX activity in leaves of maize seedlings under saline and low P stress. Each value of data represents the mean of three independent experiments, each replicated three times. Values within a column with different letters are significant at *p* ≤ 0.05 applying the LSD test.

Source of Variation	O_2_^•−^ (nmol g^−1^ FW min^−1^)	H_2_O_2_ (µmol g^−1^ FW)	MDA (nmol g^−1^ FW))	LOX (µmol min^−1^ mg^−1^ Protein)
Treatment				
Control (C)	2.93 ^d^	5.91 ^d^	24.1 ^f^	13.3 ^e^
Saline (S)	6.77 ^a^	11.8 ^b^	44.0 ^cd^	31.0 ^bc^
S + Tre	3.77 ^c^	8.63 ^c^	34.0 ^e^	22.6 ^d^
Low P (LP)	5.00 ^b^	11.8 ^b^	51.1 ^b^	34.2 ^b^
LP + Tre	3.74 ^c^	7.05 ^d^	42.3 ^d^	26.8 ^cd^
S + LP	7.28 ^a^	15.4 ^a^	64.7 ^a^	47.1 ^a^
S + LP + Tre	5.27 ^b^	10.9 ^b^	48.4 ^bc^	31.7 ^b^
SE	0.35	0.58	3.03	2.19
*F ratio (df = 6, 112)*	43.5	62.3	36.2	45.2
Variety				
BHM-7	4.73 ^b^	9.78 ^b^	52.9 ^a^	31.5 ^a^
BHM-9	5.19 ^a^	10.64 ^a^	35.3 ^b^	27.5 ^b^
SE	1.90	0.31	1.62	1.18
*F ratio (df = 1, 112)*	6.17	7.73	117.7	
Interaction				
C × BHM-7	2.84	6.2	25.5 ^g^	14.9
C × BHM-9	3.02	5.7	22.8 ^g^	11.8
S × BHM-7	7.16	11.5	53.4 ^c^	33.9
S × BHM-9	6.37	12.1	34.6 ^d-f^	28
S + Tre × BHM-7	3.37	8.2	40.9 ^d^	22.6
S + Tre × BHM-9	4.16	9	27.2 ^fg^	22.6
LP × BHM-7	4.86	10.7	66.1 ^ab^	36.2
LP × BHM-9	5.13	12.9	36.1 ^de^	32.1
LP + Tre × BHM-7	3.48	7.1	54.6 ^c^	30.1
LP + Tre × BHM-9	3.99	7	29.9 ^eg^	23.5
S + LP × BHM-7	6.52	14.6	71.7 ^a^	52
S + LP × BHM-9	8.03	16.2	57.7 ^bc^	42.2
S + LP + Tre × BHM-7	4.89	10.2	57.9 ^bc^	30.7
S + LP + Tre × BHM-9	5.65	11.6	38.9 ^d^	32.6
SE	0.98	0.83	4.28	3.11
*F ratio (df= 6, 112)*	2.05	1.39	4.14	1.65

**Table 5:** Effect of Tre on activities of SOD (Unit min^−1^ mg^−1^ protein), POD (µmol min^−1^ mg^−1^ protein), CAT (µmol min^−1^ mg^−1^ protein), GPX (nmol min^−1^ mg^t1^ protein), APX (µmol min^−1^ mg^−1^ protein), GR (nmol min^−1^ mg^−1^ protein), MDHAR (nmol min^−1^ mg^−1^ protein), DHAR (nmol min^−1^ mg^−1^ protein), and GST (nmol min^−1^ mg^−1^ protein) activities in leaves of maize seedlings under saline and low P stress. Each value of data represents the mean of three independent experiments, each replicated three times. Values within a column with different letters are significant at *p* ≤ 0.05 applying the LSD test.

Source of Variation	SOD	POD	CAT	GPX	APX	GR	MDHAR	DHAR	GST
Treatment									
Control (C)	82.2 ^cd^	0.45 ^ef^	29.1 ^cd^	76.4 ^d^	0.69 ^c^	48.6 ^c^	38.8 ^a^	99.9b	97.1 ^d^
Saline (S)	94.9 ^ab^	0.61 ^bc^	33.3 ^bc^	97.8 ^b^	0.85 ^b^	56.3 ^b^	27.9 ^d^	138.1 ^a^	136.9 ^b^
S + Tre	103.2 ^a^	0.55 ^cd^	40.4 ^a^	116.5 ^a^	1.05 ^a^	64.4 ^a^	39.4 ^a^	154.5 ^a^	161.1 ^a^
Low P (LP)	88.4 ^bc^	0.75 ^a^	32.3 ^bc^	96.7 ^bc^	0.87 ^b^	51.2 ^bc^	30.4 ^cd^	76.9 ^cd^	147.0 ^ab^
LP + Tre	95.5 ^ab^	0.64 ^b^	36.5 ^ab^	90.8 ^bc^	1.02 ^a^	49.8 ^c^	33.7 ^bc^	104.0 ^b^	159.3 ^a^
S + LP	59.8 ^e^	0.48 ^de^	26.8 ^d^	59.2 ^e^	0.53 ^d^	30.0 ^e^	29.2 ^cd^	71.1 ^d^	110.0 ^cd^
S + LP + Tre	72.4 ^d^	0.40 ^f^	31.9 ^bc^	84.7 ^cd^	0.79 ^bc^	38.4 ^d^	35.8 ^ab^	94.1 ^d^	118.0 ^c^
SE	5.38	0.04	2.45	6.28	0.06	2.96	2.57	8.89	8.29
*F ratio (df = 6, 112)*	15.6	21.7	6.80	16.1	20.5	29.1	6.40	23.8	18.1
Variety									
BHM-7	88.7 ^a^	0.57 ^a^	36.4 ^a^	89.4 ^a^	0.91 ^a^	51.8 ^a^	38.5 ^a^	105.9 ^a^	137.9 ^a^
BHM-9	81.7 ^b^	0.55 ^a^	29.4 ^b^	88.3 ^a^	0.74 ^b^	44.9 ^b^	27.7 ^b^	105.2 ^a^	127.7 ^b^
SE	2.88	0.02	1.31	3.41	0.03	1.59	1.38	4.75	4.43
*F ratio (df = 1, 112)*	6.04	0.97	28.3	0.10	31.4	18.9	51.4	0.02	5.33
Interaction									
C × BHM-7	107	0.49 ^d-f^	45.3	76.2 ^de^	1.27 ^a^	54.9	46.1 ^a^	118.9 ^c-e^	107
C × BHM-9	85.1	0.40 ^fg^	33.2	76.6 ^de^	0.83 ^c-f^	42.4	32.3 ^d-f^	80.9 ^gh^	85.1
S × BHM-7	103	0.57 ^cd^	37.4	96.6 ^bc^	1.09 ^b^	58.4	45.3 ^a^	128.2 ^b-d^	103
S × BHM-9	84.7	0.65 ^bc^	31.3	98.9 ^bc^	0.78 ^d-g^	54.2	29.4 ^d-g^	148.0 ^ab^	84.7
S+Tre × BHM-7	100	0.51 ^de^	36.9	124.0 ^a^	0.95 ^bc^	69.1	42.4 ^ab^	142.0 ^bc^	100
S+Tre × BHM-9	79.8	0.59 ^cd^	30.5	109.0 ^ab^	0.75 ^e-g^	59.7	29.1 ^d-g^	167.0 ^a^	79.8
LP × BHM-7	99.3	0.75 ^ab^	36.4	108.0 ^ab^	0.92 ^cd^	53.5	39.9 ^a-c^	81.6 ^gh^	99.3
LP × BHM-9	73.1	0.76 ^a^	29.7	85.3 ^cd^	0.68 ^f-h^	48.9	27.5 ^e-g^	72.2 ^h^	73.1
LP+Tre × BHM-7	91.7	0.71 ^ab^	35.5	83.0 ^cd^	0.90 ^c-e^	49.5	35.6 ^b-d^	108.0 ^d-f^	91.7
LP+Tre × BHM-9	71.7	0.58 ^cd^	27.4	98.6 ^bc^	0.63 ^g-i^	50.1	26.8 ^e-g^	100.0 ^e-g^	71.7
S+LP × BHM-7	89.9	0.51 ^de^	35.5	54.9 ^f^	0.88 ^c-e^	33.8	33.9 ^c-e^	73.8 ^h^	89.9
S+LP × BHM-9	61.6	0.46 ^e-g^	25	63.4 ^ef^	0.59 ^hi^	26.3	26.4 ^fg^	68.4 ^h^	61.6
S+LP+Tre × BHM-7	88	0.42 ^e-g^	34.2	83.1 ^cd^	0.87 ^c-e^	43.7	32.7 ^c-f^	88.5 ^f-h^	88
S+LP+Tre × BHM-9	57.9	0.38 ^g^	22.2	86.2 ^cd^	0.47 ^i^	33	22.8 ^g^	99.6 ^e-g^	57.9
SE	7.71	0.05	3.45	7.02	0.08	4.19	3.64	12.6	11.7
*F ratio (df = 6, 112)*	0.35	2.41	0.76	2.45	3.01	0.65	2.80	2.92	0.42

**Table 6:** Effect of Tre on MG content and activities of Gly-I and Gly-II in leaves of maize seedlings under saline and low P stress. Each value of data represents the mean of three independent experiments, each replicated three times. Values within a column with different letters are significant at *p* ≤ 0.05 applying the LSD test.

Source of Variation	MG (µmol g^−1^ FW)	Gly-I (µmol min^−1^ mg^−1^ Protein)	Gly-II (µmol min^−1^ mg^−1^ Protein)
Treatment			
Control (C)	6.50 ^d^	0.718 ^b^	0.083 ^bc^
Saline (S)	13.7 ^b^	0.706 ^b^	0.077 ^c^
S + Tre	10.6 ^c^	0.861 ^a^	0.094 ^a^
Low P (LP)	15.3 ^b^	0.567 ^c^	0.076 ^c^
LP + Tre	9.3 ^c^	0.755 ^b^	0.090 ^ab^
S + LP	19.2 ^a^	0.562 ^c^	0.047 ^e^
S + LP + Tre	14.3 ^b^	0.603 ^c^	0.061 ^d^
SE	0.88	0.04	0.004
*F ratio (df = 6, 112)*	45.6	14.2	25.2
Variety			
BHM-7	12.7 ^a^	0.596 ^b^	0.075 ^a^
BHM-9	12.6 ^a^	0.767 ^a^	0.076 ^a^
SE	0.47	0.02	0.002
*F ratio (df = 1, 112)*	0.001	59.64	0.50
Interaction			
C × BHM-7	6.54	0.641 ^cd^	0.084
C × BHM-9	13.7	0.529 ^d^	0.068
S × BHM-7	10.6	0.678 ^c^	0.096
S × BHM-9	15.3	0.569 ^cd^	0.080
S + Tre × BHM-7	9.32	0.629 ^cd^	0.091
S + Tre × BHM-9	19.2	0.555 ^d^	0.042
LP × BHM-7	14.3	0.576 ^cd^	0.063
LP × BHM-9	6.53	0.795 ^b^	0.081
LP + Tre × BHM-7	13.6	0.884 ^b^	0.085
LP + Tre × BHM-9	10.6	1.044 ^a^	0.091
S + LP × BHM-7	15.2	0.566 ^cd^	0.072
S + LP × BHM-9	9.32	0.881 ^b^	0.089
S+LP +Tre × BHM-7	19.1	0.569 ^cd^	0.051
S +LP+Tre × BHM-9	14.3	0.631 ^cd^	0.059
SE	1.25	0.06	0.006
*F ratio (df = 6, 112)*	0.0002	9.19	2.00

## Supplementary Materials

The following are available online at https://www.mdpi.com/2223-7747/8/12/568/s1, Figure S1: Effect Trehalose (Tre, 10 mM) on P deficiency symptom in maize seedlings of BHM-7 and BHM-9 under salinity (S, 150 mM) and low P (5 µM KH_2_PO_4_) stress. Seedlings were imposed stress for fifteen days.
