# Supplementary material for: Trehalose Protects Maize Plants from Salt Stress and Phosphorus Deficiency
Source: Plants (Basel). 2019 Dec 4;8(12):568. doi: 10.3390/plants8120568 (PMC6963808; doi:10.3390/plants8120568)
Supplement: Supplementary file 1 [file plants-08-00568-s001.pdf]

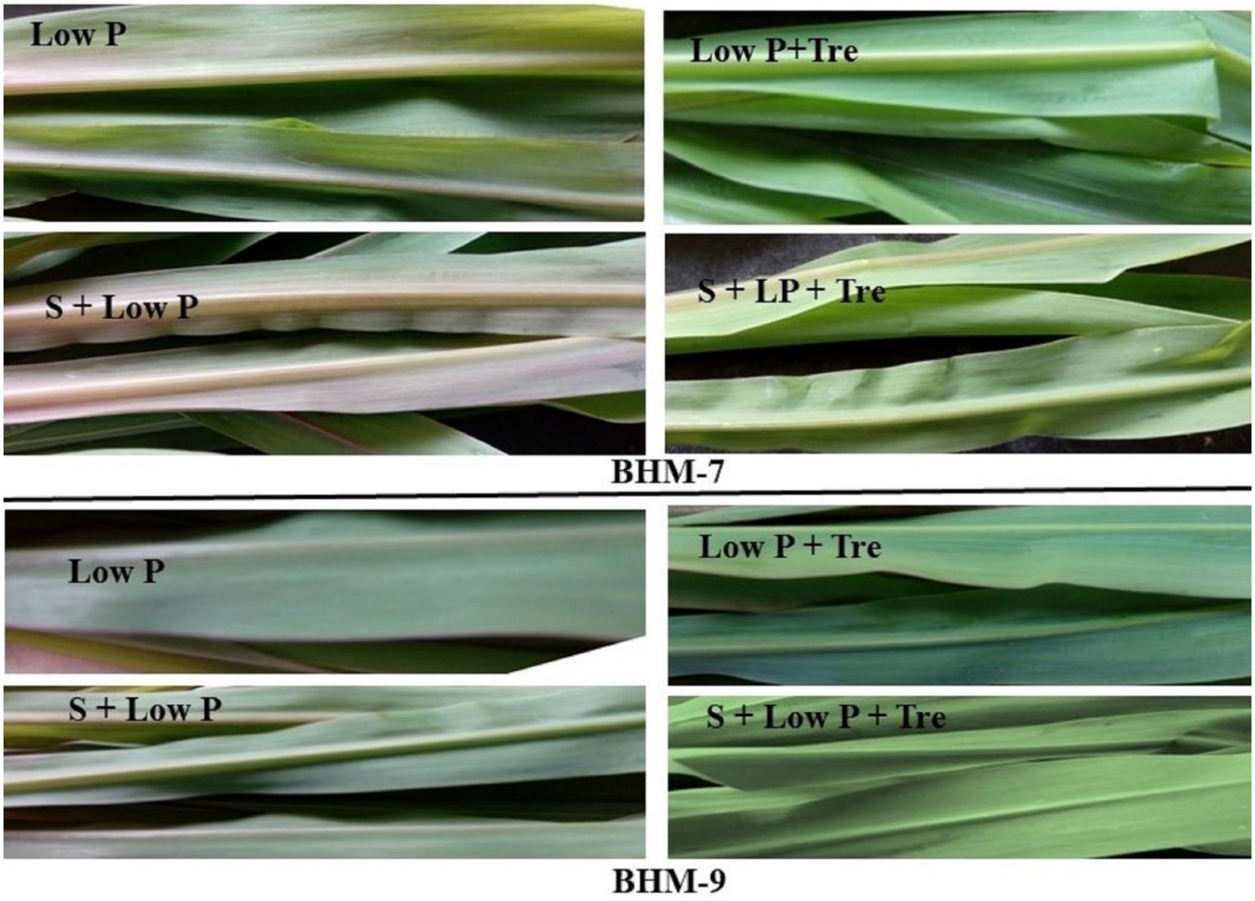

Supplementary Fig. 1. Effect of trehalose (Tre, 10 mM) on P deficiency symptom in maize seedlings of BHM-7 and BHM-9 under salinity (S, 150 mM) and low P (5 µM KH_2PO_4) stress. Seedlings were imposed stress for fifteen days
